# Selective inhibitors for JNK signalling: a potential targeted therapy in cancer

**DOI:** 10.1080/14756366.2020.1720013

**Published:** 2020-01-29

**Authors:** Qinghua Wu, Wenda Wu, Vesna Jacevic, Tanos C. C. Franca, Xu Wang, Kamil Kuca

**Affiliations:** a College of Life Science, Yangtze University, Jingzhou, China; b College of Veterinary Medicine, Nanjing Agricultural University, Nanjing, China; c Department of Chemistry, Faculty of Science, University of Hradec Kralove, Hradec Kralove, Czech Republic; d National Poison Control Centre, Military Medical Academy, Belgrade, Serbia; e Medical Faculty of the Military Medical Academy, University of Defence, Belgrade, Serbia; f Laboratory of Molecular Modeling Applied to the Chemical and Biological Defense, Military Institute of Engineering, Rio de Janeiro, Brazil; g National Reference Laboratory of Veterinary Drug Residues (HZAU) and MAO Key Laboratory for Detection of Veterinary Drug Residues, Huazhong Agricultural University, Wuhan, China

**Keywords:** JNK, selective inhibitors, cancer, tumour, SP60012, cancer therapy

## Abstract

c-Jun N-terminal kinase (JNK) signalling regulates both cancer cell apoptosis and survival. Emerging evidence show that JNK promoted tumour progression is involved in various cancers, that include human pancreatic-, lung-, and breast cancer. The pro-survival JNK oncoprotein functions in a cell context- and cell type-specific manner to affect signal pathways that modulate tumour initiation, proliferation, and migration. JNK is therefore considered a potential oncogenic target for cancer therapy. Currently, designing effective and specific JNK inhibitors is an active area in the cancer treatment. Some ATP-competitive inhibitors of JNK, such as SP600125 and AS601245, are widely used *in vitro*; however, this type of inhibitor lacks specificity as they indiscriminately inhibit phosphorylation of all JNK substrates. Moreover, JNK has at least three isoforms with different functions in cancer development and identifying specific selective inhibitors is crucial for the development of targeted therapy in cancer. Some selective inhibitors of JNK are identified; however, their clinical studies in cancer are relatively less conducted. In this review, we first summarised the function of JNK signalling in cancer progression; there is a focus on the discussion of the novel selective JNK inhibitors as potential targeting therapy in cancer. Finally, we have offered a future perspective of the selective JNK inhibitors in the context of cancer therapies. We hope this review will help to further understand the role of JNK in cancer progression and provide insight into the design of novel selective JNK inhibitors in cancer treatment.

## 1. Introduction

c-Jun N-terminal kinase (JNK) is a subfamily of mitogen-activated protein kinases (MAPK), which regulates important cellular activities, including cell proliferation, differentiation, and apoptosis^1^. There are three differently spliced genes of JNK, namely, JNK1, JNK2, and JNK3. JNK1 and JNK2 are ubiquitously expressed in most tissues, whereas JNK3 expression is predominantly in brain, and, to a lesser extent, in the heart and testis^2^. JNK signalling plays important functions in neurodegenerative diseases, inflammation, and cancer progression. They can be activated by multiple and diverse stimuli leading to varied and seemingly contradictory cellular responses^3^^,^^4^. Currently, emerging evidence indicates that JNK can regulate both cancer cell apoptosis and survival^1^^,^^5^. Earlier researchers observed that sustained activation of JNK is associated with apoptosis, whereas acute and transient activation of JNK is involved in cell proliferation or survival pathways^6^^,^^7^. The different functions of JNK may be mediated by the specific substrate or related to temporal aspects^5^.

JNK promotes tumour development in many cancers including human pancreatic cancer^8^^,^^9^, lung cancer^10^^,^^11^, breast cancer^12^^,^^13^, and skin cancer^14^. JNK is also seen to have a pro-survival role in B-lymphoma and osteosarcoma^15^^,^^16^. The pro-survival function of JNK is related to its capacity to induce cancer cell proliferation^17^, migration^18^^,^^19^, and invasion^20^. JNK is also involved in tumour initiation, as demonstrated in non-small cell lung cancer (NSCLC) cells^11^. It is required for the tumour-initiating properties of the acquired chemoresistant cancer cell lines K562/A02 and KB/VCR^21^. JNK is therefore considered a potential target for cancer therapy. Data indicate that transiently activated JNK upregulates antiapoptotic gene expression and blocks caspase activation, thereby promoting cell survival^22^. Crosstalk between JNK and other pathways is critical for cancer programming. Nuclear factor kappa B (NF-κB), p38, and JNK share common upstream activators and may act synergistically to regulate cancer cell survival^23^^,^^24^. Certain signals suppress JNK-mediated apoptosis and induce the cell survival function of JNK^25^. The role of JNK in promoting cancer cell survival involves autophagy^26^^,^^27^, as JNK can induce autophagy to counteract apoptosis^28^. Extensive evidence supports that JNK-mediated pro-survival autophagy promotes cancer cell resistance to chemotherapy^29^^,^^30^ and recent studies suggest the involvement of tumour immune evasion in this process^31^^,^^32^. JNK is closely related to traditional immune evasion regulatory factors such as transforming growth factor-β (TGF-β) and interferon-γ (IFN-γ)^33^^,^^34^. In addition, a compensatory cell proliferation mechanism underlies the regulation of JNK-mediated cancer cell survival^35–37^ .These studies highlight the complexity of the mechanisms by which JNK modulates cancer cell survival.

JNK signalling is apparently involved in cancer development and progression. Therefore, JNK is an attractive target for therapeutic intervention with small molecule kinase inhibitors. Actually some ATP-competitive (e.g. SP600125) and ATP-non-competitive inhibitors have been developed; however, some limitations are noted in these inhibitors. For example, cell toxicity and a lack of specificity are observed due to their indiscriminately inhibition of the phosphorylation of all JNK substrates^38^^,^^39^. During the last decade, some JNK inhibitors have been tested in many clinical trials. CC-401 is a seconded generation of ATP competitive inhibitors of JNK. This compound shows a high capacity in the inhibition of JNK and is also seen to show efficacy in renal injury models. However, a phase I clinical trial using CC-401 for acute myeloid leukaemia was discontinued^38^. Designing new selective JNK inhibitors is a very active area in the field of cancer therapies. Currently, some potential selective JNK inhibitors, including PYC98and PYC71N has been developed and are seen to show promising effect in the selective inhibition of one specific JNK isoform^40^^,^^41^.

In this review, we primarily summarise the function of JNK in cancer cell survival; our main goal was to mainly discuss the development of JNK inhibitors and especially the selective JNK inhibitors. Finally, we also cast a future perspective of the selective JNK inhibitors in cancer treatment. We hope this review will help to further understand the function of JNK in cancer development and provide some new lights on the development of selective JNK inhibitors in the cancer therapy.

## 2. JNK signalling activation promotes cancer cell survival

Studies indicate that JNK is a mediator of cancer cell apoptosis and cell death. JNK activation promotes gastric cancer cell apoptosis^42^^,^^43^. TNF-α-mediated caspase-8 cleavage and apoptosis are dependent on JNK activation^44^. However, emerging evidence showed that JNK conducts an antiapoptotic role of in the regulation of cancer cell survival^22^^,^^29^^,^^45^. JNK inhibits the apoptosis of vestibular schwannoma-and lymphoma cells through limiting reactive oxygen species (ROS) accumulation^46^^,^^47^. JNK regulates leukemic cell survival in response to temperature-induced stress. At 37 °C, JNK is necessary for leukemic cell survival and drug resistance; however, JNK is also involved in cold stress-induced cell death, suggesting a dual role of JNK in cell survival^45^.

JNK activation is involved in tumorigenesis in liver-, breast-, and skin cancers, brain tumours, leukaemia, multiple myeloma, and lymphoma^48^. JNK exerts a pro-survival effect by modulating cancer cell proliferation, migration, and invasion^49^^,^^50^. JNK promotes human pancreatic cancer cell proliferation by regulating microRNA-92a and GRP78^8^^,^^17^. Cancerous inhibitor of PP2A is an oncoprotein that activates MKK4/7-JNK signalling, thereby contributing to lung cancer cell proliferation^10^. Chemokine ligand-7 and CC chemokine receptor-3-correlated JNK activation are involved in the induction of metastasis in colon cancer cells^51^. In addition, the JNK/c-Jun pathway increases the invasiveness of triple negative breast cancer (TNBC) cells^20^. Activation of JNK/c-Jun is also required for epoxyeicosatrienoic acid-induced proliferation, survival, and angiogenesis in pulmonary artery endothelial cells^52^. JNK increases A549 and ovarian cancer cell migration and invasion via the hypoxia-induced activation of the 37 kDa laminin receptor precursor and IL-33/ST2 axis^19^^,^^53^. JNK is also involved in IL-33-mediated colon cancer cell stemness and macrophage recruitment^54^. JNK/Slug signalling is regulated by juxtaposed with another zinc finger protein 1 to promote prostate cancer progression^55^. Also, JNK is emerging as a pivotal regulator of tumour initiation^50^^,^^56^. For example, ectopic JNK controls the tumour-initiating capacity of NSCLC cells^11^; JNK is specifically required for maintenance of the tumour-initiating population rather than for proliferation and survival of the entire cell population.

A unique pro-survival role of JNK was demonstrated in a B-lymphoma model. Blocking the JNK pathway inhibits the proliferation of murine and human B-lymphoma cells. JNK inhibition downregulates the early growth response gene-1 (Egr-1) protein and Egr-1 overexpression partially rescues lymphoma cell apoptosis^15^. JNK may act via Egr-1, which is important for B-lymphoma survival and growth. In diffuse large B cell lymphoma, dual specificity phosphatase 4 deficiency induces constitutively active JNK signalling and contributes to tumour cell survival^57^.

JNK1 acts synergistically with Bcl-2 to promote prolonged cell survival in the absence of IL-3 or in response to different stresses^58^. The anticancer drug bortezomib activates JNK signalling leading to Bcl-2 phosphorylation and autophagy^47^. JNK and autophagy activation play a pro-survival role in this context and their inhibition increases the cytotoxic effects of bortezomib in PEL cells. JNK-mediated survival signalling also involves the transcription factor JunD^59^, as the JNK/JunD pathway collaborates with NF-κB to upregulate the expression of the antiapoptotic gene *cIAP-2*. In the absence of activated NF-κB, the JNK pathway promotes an apoptotic response^60^.

**Figure 1.**
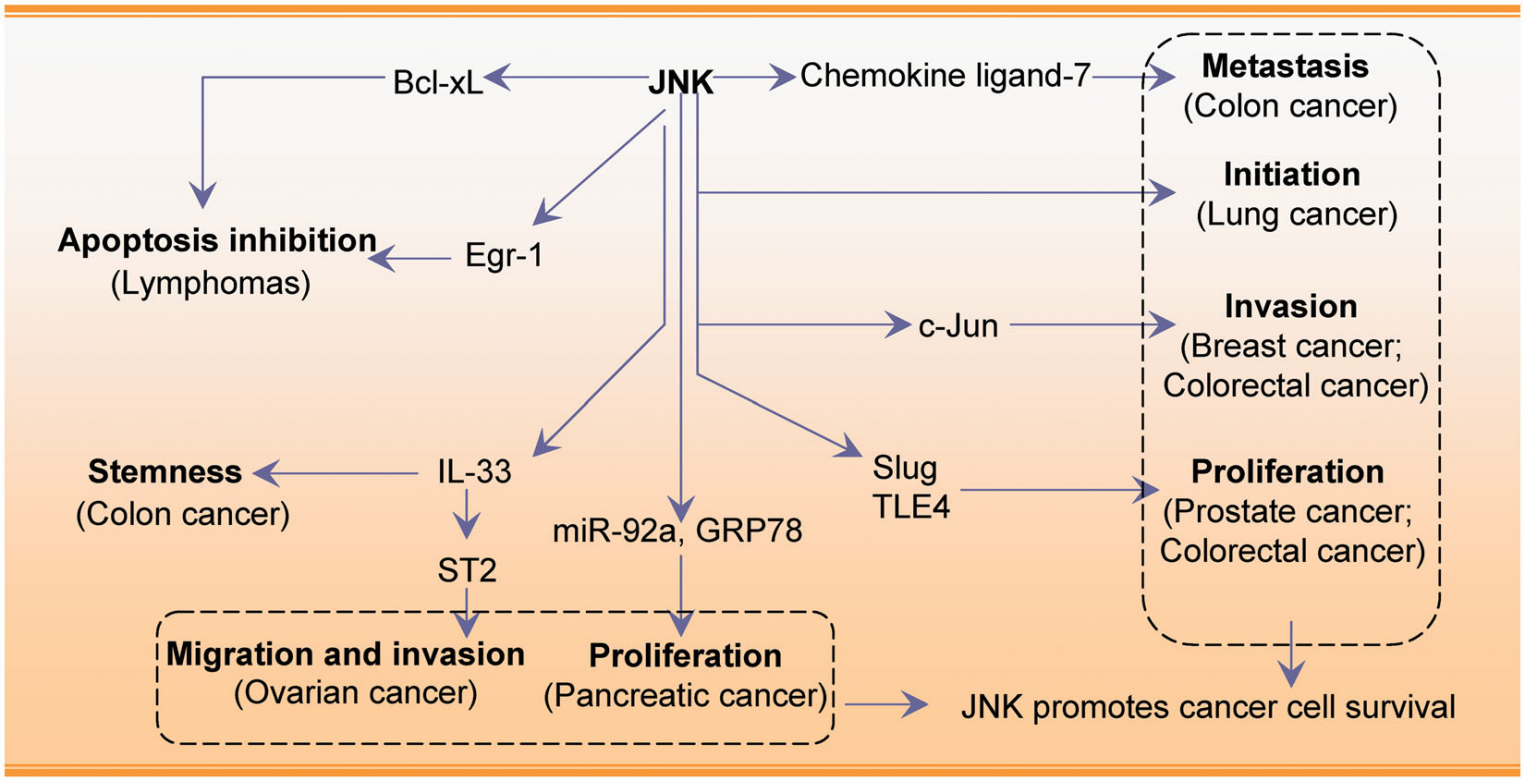
JNK promotes cancer cell survival by modulating cancer cell initiation, invasion, proliferation, and migration.

Together, JNK activation can promote cancer cell survival and plays an important role in cancer cell proliferation, migration, and invasion. JNK associates with other regulators, including MKK, c-Jun, Slug, and Egr-1 and this interaction promotes cancer cell survival. JNK is therefore a potential oncogenic target for cancer treatment. However, these findings are mostly based on *in vitro* studies and additional *in vivo* studies using animal cancer models are necessary to clarify the function of JNK. In summary, the tumour suppressive or oncogenic role of JNK is likely to depend on cancer/cell type-specific differences, the tumour microenvironment, and crosstalk with other signalling pathways. The molecular profiles associated with the role of JNK in promoting cancer cell survival are shown in Figure 1.

## 3. A “break the brake” model of JNK in cancer cell survival

Earlier studies indicate that the cancer cell survival or death function of JNK depends on functional time. In 2002, Lin^61^ proposed that a potential mechanism underlying JNK-mediated cell survival is the time course of JNK activation. Normally, transient JNK activation is important for mediating a survival response in TNF-treated cells, whereas chronic JNK activation contributes to apoptotic responses^59^^,^^61^^,^^62^. Tang et al.^63^ further showed that activation of JNK by TNF-α is transient in TNF-α-insensitive cells, whereas it is sustained in sensitive cells (apoptosis). Conversion of JNK activation from prolonged to transient suppresses TNF-α-induced apoptosis (survival)^63^. Ventura et al.^6^ also showed that the time course of JNK signalling can influence the biological response to JNK activation. These authors demonstrated that the early transient phase of JNK activation (<1 h) promotes cell survival, whereas the later and more sustained phase of JNK activation (1–6 h) mediates proapoptotic signalling. The dependency of the survival response on early JNK activation is attributed to antiapoptotic gene expression at an early period. Indeed, transient JNK1 activation (<2 h) cross-talks with STAT3 to upregulate the antiapoptotic genes Bcl-2 and Bcl-xL, and inhibit the proapoptotic gene Bax, thereby promoting RAW264.7 cell survival^64^.

**Figure 2.**
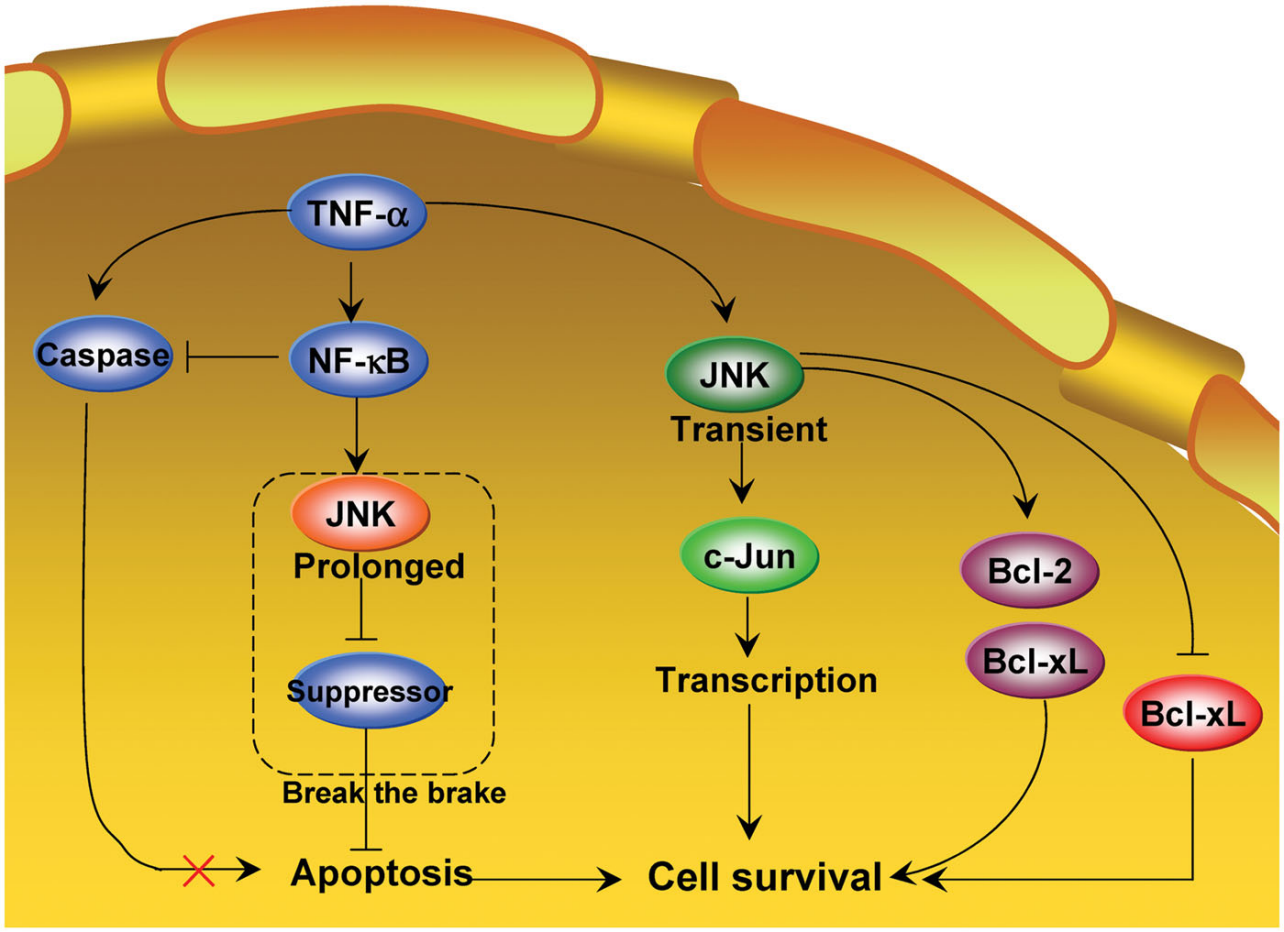
The “break the brake” hypothesis of JNK during regulation of cancer cell survival.

Lin^61^ further hypothesised that prolonged JNK activation may “break the brake” on apoptosis. In this model (Figure 2), JNK may be a modulator rather than an intrinsic component of the apoptotic machinery. JNK activation thus facilitates, but does not induce, apoptosis. Prolonged JNK activation may inhibit apoptotic pathway suppressors in the presence of TNF-α, thereby allowing apoptotic cells to fulfil their death wish^22^. TNF-α induces apoptosis in TNF-α-sensitive cells^63^. While caspase activation initiates and executes apoptosis, prolonged JNK activation promotes apoptosis by inactivating suppressors of the mitochondrial-dependent death pathway. Activation of NF-κB by TNF-α blocks caspase activation and prevents prolonged JNK activation, thereby inhibiting TNF-α-induced apoptosis^63^. However, additional evidence is necessary to prove this hypothesis, as the “break the brake” model has not been tested since its proposal 15 years ago.

## 4. JNK inhibitors in the treatment of cancer

Due to the important role of JNK in the cancer development, designing effective and specific JNK inhibitors is a very active filed of research in different academic and industrial laboratories in the world. Currently, the clinical success of selective kinase inhibitors, such as imatinib and erlotinib, as therapeutic agents for several human cancers has prompted substantial interest in the development and clinical testing of such inhibitors for a wide variety of malignancies^65^^,^^66^. Protein kinase inhibitors are widely used as therapeutic agents in many cancers; however, they have potential off-target effects associated with the highly conserved protein kinase family^65^^,^^67^.

SP600125that belongs to the ATP-competitive inhibitor, is the most commonly used JNK inhibitor in many *in vitro* and *in vivo* studies^68^^,^^69^. This compound has remarkable antitumor potential in different cancers, including stomach cancer, oral squamous cell carcinoma, lung adenocarcinoma, cholangiocarcinoma, colon carcinoma, pancreatic cancer, and glioblastoma^65^^,^^70^. AS601245 is a cell-permeable JNK inhibitor that shows promising anticancer effects in colon cancer and T cell acute lymphoblastic leukaemia^71^^,^^72^. Another promising JNK inhibitor is JNK-IN-8, which can sensitise TNBC cells to lapatinib by upregulating p65 and Nrf2. Combination treatment significantly increased the survival of mice bearing MDA-MB-231 human TNBC xenografts^73^^,^^74^. Mice treated with SP600125 or JNK-IN-8 showed significantly increased survival rates after invasive fungal infections, particularly with Candida albicans^75^. AS602801 is cytotoxic against CSCs derived from human pancreatic cancer, NSCLC, ovarian cancer, and glioblastoma^76^. AS602801 also inhibits the self-renewal and tumour-initiating capacity of CSCs. *In vivo*, AS602801 inhibits CSCs in established xenograft tumours when administered at a dose and schedule that does not adversely affect the health of tumour-bearing mice^76^. However, as is known, these inhibitors have varying toxicity and lacks specificity as they indiscriminately inhibit phosphorylation of all JNK substrates^38^.

Hepatic ischemia/reperfusion (I/R), which is characterised by severe inflammation and cell death, causes significant liver damage and hepatic cancer^77^. Ginsenoside Rg1 (20 mg/kg/day), which inhibits JNK signalling, significantly promotes hepatic function and suppresses liver necrosis and inflammatory responses^78^. An azaquinolone analog168 and N-alkyl (propyl and butyl)-bearing pyrazoloanthrone scaffolds show promise as therapeutic inhibitors of JNK^79^. Angell et al.^80^ and Jang et al.^81^ further showed that N-(3-cyano-4,5,6,7-tetrahydro-1-benzothien-2-yl)amides acts as an ATP-binding site-targeting inhibitor of JNK2 and JNK3. The mechanism was further studied, and the authors showed that this inhibitor can cause apoptotic DNA fragmentation in parallel with G2/M arrest, phosphorylation of Bcl-2, Mcl-1, and Bim and activation of Bak and the caspase cascade, suggesting its anticancer potential^81^.

However, JNK has at least three isoforms (JNK1–3) with different functions in cancer development. These inhibitors lack specificity and selectivity for the different JNK isoforms^65^. Suppressing total JNK activity is not an appropriate strategy because different JNK isoforms have distinct functions in cancer, asthma, diabetes, or Parkinson’s disease^82^. Apparently, the identification and development of selective JNK inhibitors should be a valuable strategy for the treatment of cancer.

**Figure 3.**
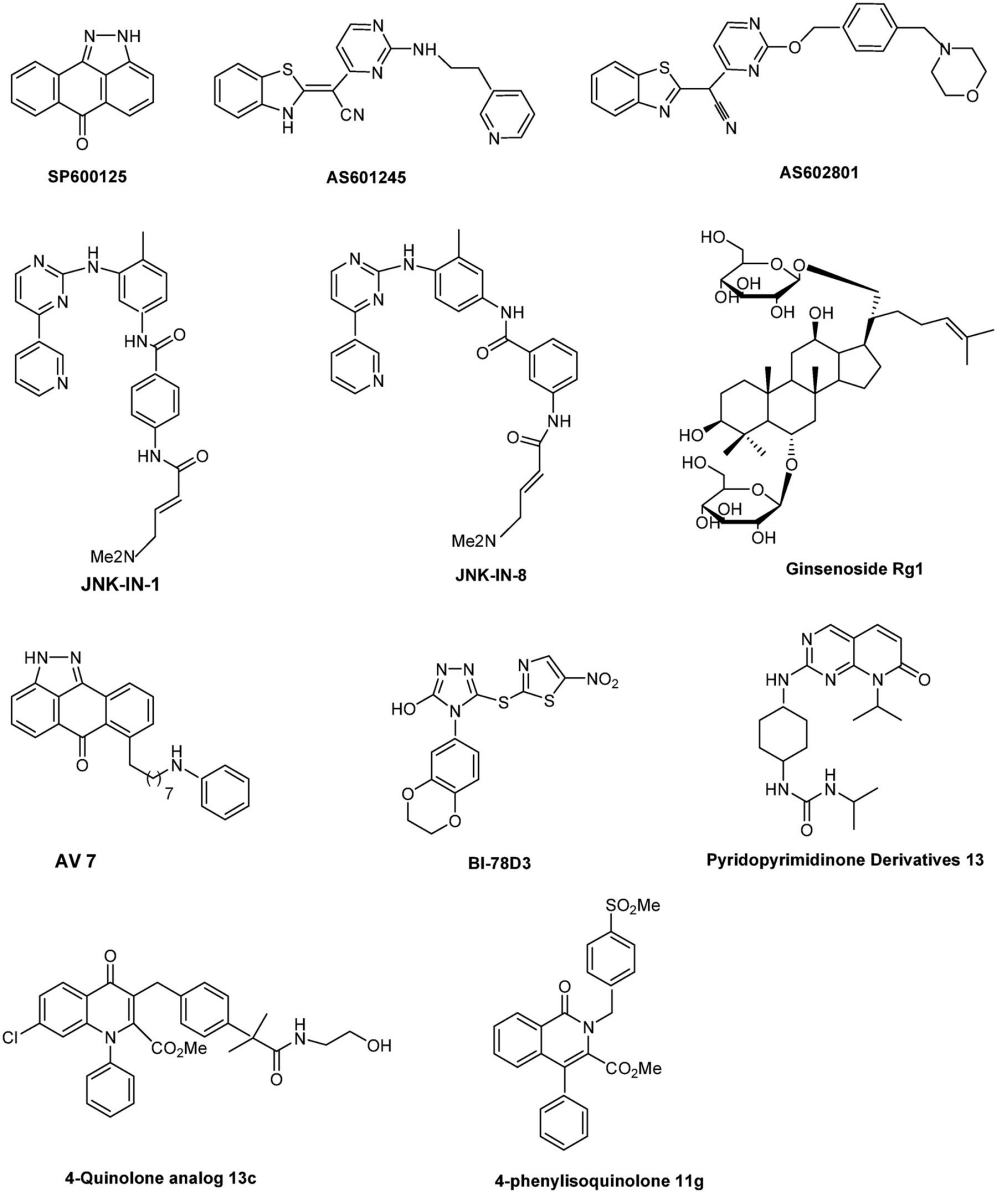
Chemical structures of selective JNK inhibitors.

Several peptide inhibitors have been developed, such as JNKi-1^83^, which has been successfully used in mouse models, for example, HCC or Bi-78D3, which inhibits JNK activity by interfering with binding to the JNK-interacting protein 1 scaffold^84^. However, these novel compounds are not selective inhibitors of JNK1–3 and isoform-specific JNK inhibitors are rarely reported. Among few isoform-specific JNK inhibitors identified, the isoquinolone derivative methylsulfonyl exhibits potent inhibitory activity against JNK1 and acts as a JNK1 selective and ATP competitive inhibitor^85^. Moreover, this inhibitor significantly inhibits cardiac hypertrophy in a rat pressure-overload model without affecting blood pressure. Yao et al.^86^ reported that 7–(6-N-phenylaminohexyl)amino-2H-anthra[1,9-cd]pyrazol-6-one (AV-7) has selective inhibitory activity against JNK1, but not JNK2/3. The novel inhibitory peptides PYC98, PYC71N, and adamantyl azaquinolone selectively inhibit JNK1 activity towards c-Jun^40^^,^^41^. Currently, many researchers and laboratories are focussing on the selective inhibition of JNK3, because this isoform is mainly expression in the brain and is an attractive target of neurodegenerative disease^38^. Pyrimidinyl-substituted benzazole-acetonitriles specifically target JNK3, showing inhibitory activity in the double-digit nanomolar range^87^. A specific JNK3 inhibitor derived from triazolone variants showed >10-fold higher selectivity than JNK1^88^. In addition, 6-anilinoindazoles^89^, 20-anilino-4,40-bipyridines^90^, isoxazole derivatives^17^, XG-102^91^, and pyridopyrimidinone derivatives^92^ were identified as selective inhibitors of JNK3. XG-102 shows potential for the treatment of patients with inflammatory bowel disease^93^. The chemical structures of these selective inhibitors are shown in Figure 3.

The studies summarised above indicate that selective JNK inhibitors are important for the treatment of cancer. However, compared with other pathway inhibitors, there are relatively less JNK inhibitors for clinical application. This could be partly attributed to our poor understanding of the complex functions of JNK, as JNK not only functions in cancer development, but also contributes to cancer cell death and is involved in many physiological processes^39^. Nevertheless, some inhibitors of JNK have been tested in the clinical study. For example, CC-401, a second-generation ATP-competitive inhibitor, shows antineoplastic activity. However, it is a pity that a Phase I clinical study of CC-401 in the acute myeloid leukaemia was discontinued and the development of CC-401 was stopped. The clinical trials of other JNK inhibitors, including CC-930, were terminated by the sponsor due to the lack of support^94^. PGL5001 (AS601245) is another ATP-completive inhibitor and is currently being evaluated in a Phase II clinical trial^38^. It should be noted that the context of tissue-dependent expression was not considered in the clinical trials of JNK inhibitors. The lack of efficacy and the side effects are highly possible, at least in part, due to the lack of selecting and identifying of a subset of patients who would respond positively to anti-JNK therapies^38^.

The role of JNK as a target for cancer treatment needs to be further explored to develop substrate-specific inhibitors, this may contribute to the alleviation of different types of cancer^48^^,^^95^. Moreover, future clinical studies should mainly consider the context of tissue-dependency too. The potential of JNK inhibitors for the treatment of cancer and other clinical applications is summarised in Table 1.

**Table 1.** Anticancer and other potential clinical applications of JNK inhibitors.

Inhibitor	Target	IC_50_	Clinical potentials	References
SP600125	JNKs	JNK1/2 = 40 nM; JNK3 = 90 nM	Anticancer potential for stomach cancer, oral squamous carcinoma, lung adenocarcinoma, cholangiocarcinoma, and colon carcinoma.	^65^
AS601245	JNKs	JNK1 = 150 nM; JNK2 = 220 nM; JNK3 = 70 nM	Anticancer potential for colon cancer and T cell acute lymphoblastic leukaemia.	^71^^,^^72^
AS602801	JNKs	JNK1 = 80 nM; JNK2 = 90 nM; JNK3 = 230 nM	Induces apoptosis of cancer stem cells.	^76^
JNK-IN-1	JNKs	JNKs = 2.31 nM	Anticancer potential for skin cancer and attenuation of chronic colitis.	^93^^,^^96^
BI-78D3	JNKs	JNKs = 280 nM	Anticancer potential for osteosarcoma.	^97^
JNK-IN-8	JNKs		Sensitizes triple-negative breast cancer cells to lapatinib.	^73^
Bi-78D3	JNKs		Blocks JNK-dependent Con A-induced liver damage; restores insulin sensitivity in mouse models of type 2 diabetes.	^84^
Ginsenoside Rg1	JNKs		Protects against ischemia/reperfusion-induced liver damage.	^98^
4-fluorophenyl isoxazoles	JNKs	JNK1 = 13 nM; JNK3 = 16 nM	A structure-activity relationship study was performed; however, the cellular potency and *in vivo* properties need to be improved.	^17^
XG-102 (D-JNKI-1)	JNKs		31-D-amino-acid peptide; it is safe for the treatment of patients with post-surgery or post-trauma intraocular inflammation; protects against TNBS-induced colitis.	^99^^,^^100^
4-quinolone analogues	JNKs	JNK1 = 62 nM; JNK2 = 170 nM	This compound shows excellent kinase selectivity and impressive efficacy in a rodent asthma model.	^101^
CC-930	JNKs	JNK1 = 61 nM; JNK2 = 7 nM; JNK3 = 6 nM	Does not inhibit CYP450 enzymes significantly; it shows low toxicity, is well tolerated, and exposure is dose-proportional.	^94^
Quinazoline	JNK3	JNK3 = 40 nM	Shows good brain penetration and pharmacokinetic (PK) properties and is a candidate for *in vivo* evaluation in the central nervous system (CNS)efficacy models.	^102^
Triazolothione 1	JNK3	JNK3 = 1.07 nM	It has CNS properties.	^88^
Pyridopyrimidinone Derivatives	JNK3	JNK3 = 15 nM	Clean CYP-450 inhibition profile; good microsomal stability; and good oral bioavailability.	^92^
AV7	JNK1		It has the potential to treat of diabetes.	^86^
D-PYC98	JNK1		A novel retro-inverso peptide; inhibits p38, and c-Jun Ser63 phosphorylation during hyperosmotic stress.	^41^
Isoquinolone derivatives	JNK1	JNK1 = 86 nM	They can be novel therapeutic agents for heart failure without affecting blood pressure.	^85^
JNK inhibitor IX (JNKi)	JNK2/3		Induce DNA fragmentation and apoptotic cell death in human Jurkat T cells.	^81^

## 5. Future perspectives of selective JNK inhibitors in cancer therapy

Development of selective JNK inhibitors that target specific JNK-mediated cellular events is challenging. Currently, numerous selective pathway inhibitors have entered the clinical and market stages as anticancer therapies. For example, STAT3 inhibitors OPB-31121 and OPB-51602 have completed Phase I and II studies for treatment of various cancers, including advanced solid tumours, non-Hodgkin’s lymphoma, and liver cancer^103^^,^^104^. Two inhibitors of the PI3K/AKT/mTOR pathway (SF1126 and GDC-0980) are in Phase I and II clinical trials, respectively^105^^,^^106^. The FDA has approved the Bruton*’*s tyrosine kinase (BTK) inhibitor ibrutinib for treatment of chronic lymphocyte leukaemia; this prompted researchers to conduct “me too/me better” investigations^107^. However, the complex roles of the JNK pathway during tumorigenesis remain unclear. To date, the exact mechanism by which JNK regulates cancer development and which isoforms play the major role in this context, are unclear. The pro-survival and apoptosis functions of JNK are not fully understood. The effects of JNK signalling on tumour cell growth are not static; the function of JNK may change according to time or cell cycle stage. Therefore, development of selective JNK inhibitors should take into account the functional time and the function of different JNK isoforms also.

Currently, an ideal JNK-specific inhibitor has not been identified yet, and many reported JNK inhibitors lack specificity for JNK with different conformations. Highly specific JNK inhibitors can be developed by chemical synthesis, by screening natural products that specifically inhibit the JNK pathways or by chemically modifying these natural products. Of course, development of JNK inhibitors for cancer therapy requires a systemic approach that requires consideration of metabolism, toxicity, drug resistance, side effects, and price. Development of substrate- or isoform-specific inhibitors and their clinical application may represent an innovative therapeutic approach to prevention and treatment of some cancers.

Development of novel JNK inhibitors specific for different tumour types should be encouraged. However, considering the dual roles of JNK in cancer, treatment with a JNK inhibitor during chemotherapy would require careful planning. It is very possible that the mechanism underlying JNK-mediated cell survival and even chemoresistance is not the same for different cancers. We think that, as a first step, we may do not need to clarify all JNK-mediated resistance mechanisms for all cancers. Rather, we can try to develop a single specific JNK inhibitor for one or some, typical cancers once we understand the JNK-mediated mechanisms relevant to that cancer. This process will make development of JNK inhibitors much faster and we believe that more specific JNK inhibitors will be tested in many clinical studies in the near future.

## 6. Conclusion

JNK signalling is a crucial oncogenic target that raises many researcher’s interest. Uncovering highly efficient selective JNK inhibitors is a hot topic of the last decade. Currently, some selective JNK inhibitors have been developed; however, more clinical studies of these inhibitors should be tested. Moreover, clinical studies of JNK inhibitors should determine which JNK inhibitor is most effective against cancer therapy. We should make further efforts to understand which JNK proteins are beneficial targets and should also uncover the potential mechanisms of the inhibitors on the various physiological process. The particular use of JNK inhibitors should be considered as the genetic background and the precise signalling pathway that directs the carcinogenic properties of the cells for a given cancer type needs to be noted. In conclusion, we believe that there is a bright future of JNK inhibitors and their use in cancer therapies should be tested in more clinical trials.

## References

[1] Gkouveris I, Nikitakis NG. Role of JNK signaling in oral cancer: a mini review. Tumour Biol 2017;39:101042831771165. 10.1177/1010428317711659 28639904

[2] Kumar A, Singh UK, Kini SG, et al. JNK pathway signaling: a novel and smarter therapeutic targets for various biological diseases. Future Med Chem 2015;7:2065–86. 26505831 10.4155/fmc.15.132

[3] Zeke A, Misheva M, Reményi A, Bogoyevitch MA. JNK signaling: regulation and functions based on complex protein-protein partnerships. Microbiol Mol Biol Rev 2016;80:793–835. 27466283 10.1128/MMBR.00043-14 PMC4981676

[4] Minero VG, Khadjavi A, Costelli P, et al. JNK activation is required for TNFα-induced apoptosis in human hepatocarcinoma cells. Int Immunopharmacol 2013;17:92–8. 23751896 10.1016/j.intimp.2013.05.017

[5] Bode AM, Dong Z. The functional contrariety of JNK. Mol Carcinog 2007;46:591–8. 17538955 10.1002/mc.20348 PMC2832829

[6] Ventura J-J, Hübner A, Zhang C, et al. Chemical genetic analysis of the time course of signal transduction by JNK. Mol Cell 2006;21:701–10. 16507367 10.1016/j.molcel.2006.01.018

[7] Dhanasekaran DN, Reddy EP. JNK-signaling: a multiplexing hub in programmed cell death. Genes Cancer 2017;8:682–94. 29234486 10.18632/genesandcancer.155 PMC5724802

[8] 15Suzuki S, Okada M, Shibuya K, et al. JNK suppression of chemotherapeutic agents-induced ROS confers chemoresistance on pancreatic cancer stem cells. Oncotarget 2014;6:458–70. 10.18632/oncotarget.2693 PMC4381607 25473894

[9] Yuan XP, Dong M, Li X, Zhou JP. GRP78 promotes the invasion of pancreatic cancer cells by FAK and JNK. Mol Cell Biochem 2015;398:55–62. 25218495 10.1007/s11010-014-2204-2

[10] Peng B, Chai Y, Li Y, et al. CIP2A overexpression induces autoimmune response and enhances JNK signaling pathway in human lung cancer. BMC Cancer 2015;15:895. 26560124 10.1186/s12885-015-1899-0 PMC4642650

[11] Okada M, Shibuya K, Sato A, et al. Specific role of JNK in the maintenance of the tumor-initiating capacity of A549 human non-small cell lung cancer cells. Oncol Rep 2013;30:1957–64. 23912840 10.3892/or.2013.2655

[12] Phelps-Polirer K, Abt MA, Smith D, Yeh ES. Co-targeting of jnk and hunk in resistant her2-positive breast cancer. Plos One 2016;11:e0153025. 27045589 10.1371/journal.pone.0153025 PMC4821489

[13] Puvirajesinghe TM, Bertucci F, Jain A, et al. Identification of p62/SQSTM1 as a component of non-canonical Wnt VANGL2-JNK signalling in breast cancer. Nat Commun 2016;7:10318. 26754771 10.1038/ncomms10318 PMC4729931

[14] Zhang JY, Selim MA. The role of the c-Jun N-terminal Kinase signaling pathway in skin cancer. Am J Cancer Res 2012; 2:691–8. 23226615 PMC3512184

[15] Gururajan M, Chui R, Karuppannan AK, et al. c-Jun N-terminal kinase (JNK) is required for survival and proliferation of B-lymphoma cells. Blood 2005;106:1382–91. 15890690 10.1182/blood-2004-10-3819 PMC1895189

[16] Li W, Zhang H, Nie A, et al. mTORC1 pathway mediates beta cell compensatory proliferation in 60% partial-pancreatectomy mice. Endocrine 2016;53:117–28. 26818915 10.1007/s12020-016-0861-5

[17] He Y, Duckett D, Chen W, et al. Synthesis and SAR of novel isoxazoles as potent c-jun N-terminal kinase (JNK) inhibitors. Bioorg Med Chem Lett 2014;24:161–4. 24332487 10.1016/j.bmcl.2013.11.052 PMC4540177

[18] Zhao HF, Wang J, Jiang HR, et al. PI3K p110β isoform synergizes with JNK in the regulation of glioblastoma cell proliferation and migration through Akt and FAK inhibition. J Exp Clin Cancer Res 2016;35:78. 27176481 10.1186/s13046-016-0356-5 PMC4866398

[19] Zhou Y, Wang Y, Zhao Z, et al. 37LRP induces invasion in hypoxic lung adenocarcinoma cancer cells A549 through the JNK/ERK/c-Jun signaling cascade. Tumour Biol 2017;39:101042831770165. 10.1177/1010428317701655 28618937

[20] Han J, Jeon M, Shin I, Kim S. Elevated STC-1 augments the invasiveness of triple-negative breast cancer cells through activation of the JNK/c-Jun signaling pathway. Oncology Rep 2016;36:1764–71. 10.3892/or.2016.4977 27459971

[21] Liu G, Jiang X, Zhu X, et al. Ros activates jnk-mediated autophagy to counteract apoptosis in mouse mesenchymal stem cells in vitro. Acta Pharmacologica Sinica 2015; 36:1473–9. 26592514 10.1038/aps.2015.101 PMC4816227

[22] Lin A, Dibling B. The true face of JNK activation in apoptosis. Aging Cell 2002; 1:112–6. 12882340 10.1046/j.1474-9728.2002.00014.x

[23] Ruan J, Qi Z, Shen L, et al. Crosstalk between JNK and NF-κB signaling pathways via HSP27 phosphorylation in HepG2 cells. Biochem Biophys Res Commun 2015;456:122–8. 25446109 10.1016/j.bbrc.2014.11.045

[24] Svensson C, Part K, Künnis-Beres K, et al. Pro-survival effects of JNK and p38 MAPK pathways in LPS-induced activation of BV-2 cells. Biochem Biophys Res Commun 2011;406:488–92. 21338578 10.1016/j.bbrc.2011.02.083

[25] Okamura T, Antoun G, Keir ST, et al. Phosphorylation of glutathione S-transferase P1 (GSTP1) by epidermal growth factor receptor (EGFR) pomotes formation of the GSTP1-c-Jun N-terminal kinase (JNK) complex and suppresses JNK downstream signaling and apoptosis in brain tumor cells. J Biol Chem 2015;290:30866–78. 26429914 10.1074/jbc.M115.656140 PMC4692215

[26] Yin H, Yang X, Gu W, et al. HMGB1-mediated autophagy attenuates gemcitabine-induced apoptosis in bladder cancer cells involving JNK and ERK activation. Oncotarget 2017;8:71642–56. 29069735 10.18632/oncotarget.17796 PMC5641078

[27] Li J, Zhao L, Zhao X, et al. Foxo1 attenuates NaF-induced apoptosis of LS8 cells through the JNK and mitochondrial pathways. Biol Trace Elem Res 2018;181:104–11. 28429284 10.1007/s12011-017-1015-1

[28] Liu GY, Jiang XX, Zhu X, et al. ROS activates JNK-mediated autophagy to counteract apoptosis in mouse mesenchymal stem cells in vitro. Acta Pharmacol Sin 2015;36:1473–9. 26592514 10.1038/aps.2015.101 PMC4816227

[29] Almasi S, Kennedy BE, El-Aghil M, et al. TRPM2 channel-mediated regulation of autophagy maintains mitochondrial function and promotes gastric cancer cell survival via the JNK-signaling pathway. J Biol Chem 2018;293:3637–50. 29343514 10.1074/jbc.M117.817635 PMC5846146

[30] Huang XL, Zhang H, Yang XY, et al. Activation of a c-Jun N-terminal kinase-mediated autophagy pathway attenuates the anticancer activity of gemcitabine in human bladder cancer cells. Anticancer Drug 2017;28:596–602. 10.1097/CAD.0000000000000499 28430744

[31] Ren K, Mo ZC, Liu X, et al. TGF-β down-regulates apolipoprotein M expression through the TAK-1-JNK-c-Jun pathway in HepG2 cells. Lipids 2017;52:109–17. 28039587 10.1007/s11745-016-4227-9

[32] Liang Y, Jiao J, Liang L, et al. TRAF6 mediated the promotion of salivary adenoid cystic carcinoma progression through Smad-p38-JNK signaling pathway induced by TGF-β. J Oral Pathol Med 2018;47:583–9. 29577454 10.1111/jop.12709

[33] Mao CP, Wu T, Song KH, Kim TW. Immune-mediated tumor evolution: Nanog links the emergence of a stem like cancer cell state and immune evasion. Oncoimmunology 2014;3:e947871. 25610734 10.4161/21624011.2014.947871 PMC4292413

[34] Abdollahi A, Folkman J. Evading tumor evasion: current concepts and perspectives of anti-angiogenic cancer therapy. Drug Resist Updat 2010;13:16–28. 20061178 10.1016/j.drup.2009.12.001

[35] Ryoo HD, Gorenc T, Steller H. Apoptotic cells can induce compensatory cell proliferation through the JNK and the wingless signaling pathways. Dev Cell 2004;7:491–501. 15469838 10.1016/j.devcel.2004.08.019

[36] Vucur M, Reisinger F, Gautheron J, et al. RIP3 inhibits inflammatory hepatocarcinogenesis but promotes cholestasis by controlling caspase-8- and JNK-dependent compensatory cell proliferation. Cell Rep 2013;4:776–90. 23972991 10.1016/j.celrep.2013.07.035

[37] Poulton JS, Cuningham JC, Peifer M. Acentrosomal drosophila epithelial cells exhibit abnormal cell division, leading to cell death and compensatory proliferation. Dev Cell 2014;30:731–45. 25241934 10.1016/j.devcel.2014.08.007 PMC4182331

[38] Messoussi A, Feneyrolles C, Bros A, et al. Recent progress in the design, study, and development of c-Jun N-terminal kinase inhibitors as anticancer agents. Chem Biol 2014; 21:1433–43. 25442375 10.1016/j.chembiol.2014.09.007

[39] Wu Q, Wu W, Fu B, et al. JNK signaling in cancer cell survival. Med Res Rev 2019;39:2082–104. 30912203 10.1002/med.21574

[40] Haynes NE, Scott NR, Chen LC, et al. Identification of an adamantyl azaquinolone JNK selective inhibitor. ACS Med Chem Lett 2012;3:764–8. 24900545 10.1021/ml300175c PMC4025727

[41] Ngoei KR, Catimel B, Church N, et al. Characterization of a novel JNK (c-Jun N-terminal kinase) inhibitory peptide. Biochem J 2011;434:399–413. 21162712 10.1042/BJ20101244

[42] Chen Y, Song S, Su W. Activation of JNK by TPA promotes apoptosis via PKC path way in gastric cancer cells. World J Gastroenterol 2002;8:1014–8. 12439916 10.3748/wjg.v8.i6.1014 PMC4656371

[43] Androutsopoulos VP, Spandidos DA. Anticancer pyridines induce G2/M arrest and apoptosis via p53 and JNK upregulation in liver and breast cancer cells. Oncol Rep 2018;39:519–24. 29207138 10.3892/or.2017.6116 PMC5783619

[44] Deng Y, Ren X, Yang L, et al. A jnk-dependent pathway is required for tnfalpha-induced apoptosis. Cell 2003;115:61–70. 14532003 10.1016/s0092-8674(03)00757-8

[45] Cerezo D, Ruiz-Alcaraz AJ, Lencina-Guardiola M, et al. Attenuated JNK signaling in multidrug-resistant leukemic cells. Dual role of MAPK in cell survival. Cell Signal 2017;30:162–70. 27940051 10.1016/j.cellsig.2016.12.003

[46] Yue WY, Clark JJ, Fernando A, et al. Contribution of persistent C-Jun N-terminal kinase activity to the survival of human vestibular schwannoma cells by suppression of accumulation of mitochondrial superoxides. Neuro Oncol 2011;13:961–73. 21697181 10.1093/neuonc/nor068 PMC3158009

[47] Granato M, Santarelli R, Lotti LV, et al. JNK and macroautophagy activ ation by bortezomib has a pro-survival effect in primary effusion lymphoma cells. PLoS One 2013;8:e75965. 24086672 10.1371/journal.pone.0075965 PMC3784388

[48] Bubici C, Papa S. JNK signalling in cancer: in need of new, smarter therapeutic targets. Br J Pharmacol 2014;171:24–37. 24117156 10.1111/bph.12432 PMC3874694

[49] Greenman C, Stephens P, Smith R, et al. Patterns of somatic mutation in human cancer genomes. Nature 2007;446:153–8. 17344846 10.1038/nature05610 PMC2712719

[50] Wagner EF, Nebreda AR. Signal integration by JNK and p38 MAPK pathways in cancer development. Nat Rev Cancer 2009;9:537–49. 19629069 10.1038/nrc2694

[51] Lee YS, Kim SY, Song SJ, Hong HK, et al. Crosstalk between CCL7 and CCR3 promotes metastasis of colon cancer cells via ERK-JNK signaling pathways. Oncotarget 2016;7:36842–53. 27167205 10.18632/oncotarget.9209 PMC5095043

[52] Ma J, Zhang L, Han W, et al. Activation of JNK/c-Jun is required for the proliferation, survival and angiogenesis induced by EETs in PAECs. J Lipid Res 2012;53:1093–105. 22493087 10.1194/jlr.M024398 PMC3351816

[53] Tong X, Barbour M, Hou K, et al. Interleukin-33 predicts poor prognosis and promotes ovarian cancer cell growth and metastasis through regulating ERK and JNK signaling pathways. Mol Oncol 2016;10:113–25. 26433471 10.1016/j.molonc.2015.06.004 PMC5528933

[54] Fang M, Li Y, Huang K, et al. IL33 promotes colon cancer cell stemness via JNK activation and macrophage recruitment. Cancer Res 2017;77:2735–45. 28249897 10.1158/0008-5472.CAN-16-1602 PMC5760170

[55] Sung Y, Park S, Park SJ, et al. Jazf1 promotes prostate cancer progression by activating JNK/Slug. Oncotarget 2017; 9:755–65. 29416651 10.18632/oncotarget.23146 PMC5787507

[56] Kitanaka C, Sato A, Okada M. JNK signaling in the control of the tumor-initiating capacity associated with cancer Stem Cells. Genes Cancer 2013;4:388–96. 24349636 10.1177/1947601912474892 PMC3863334

[57] Schmid CA, Robinson MD, Scheifinger NA, et al. DUSP4 deficiency caused by promoter hypermethylation drives JNK signaling and tumor cell survival in diffuse large B cell lymphoma. J Exp Med 2015; 212:775–92. 25847947 10.1084/jem.20141957 PMC4419353

[58] Deng X, Xiao L, Lang W, et al. Novel role for JNK as a stress-activated Bcl2 kinase. J Biol Chem 2001;276:23681–8. 11323415 10.1074/jbc.M100279200

[59] Lamb JA, Ventura JJ, Hess P, et al. JunD mediates survival signaling by the JNK signal transduction pathway. Mol Cell 2003;11:1479–89. 12820962 10.1016/s1097-2765(03)00203-x

[60] Himes SR, ester DP, Ravasi T, et al. The JNK are important for development and survival of macrophages. J Immunol 2006;176:2219–28. 16455978 10.4049/jimmunol.176.4.2219

[61] Lin A. Activation of the JNK signaling pathway: breaking the brake on apoptosis. Bioessays 2003;25:17–24. 12508278 10.1002/bies.10204

[62] Lin A. A five-year itch in TNF-alpha cytotoxicity: the time factor determines JNK action. Dev Cell 2006; 10:277–8. 16516830 10.1016/j.devcel.2006.02.006

[63] Tang F, Tang G, Xiang J, et al. Absence of NF-kB-mediated inhibition of JNK activation contributes to TNF-a induced apoptosis in MCF-7 cells. Mol Cell Biol 2002;22:8571–9. 12446776 10.1128/MCB.22.24.8571-8579.2002 PMC139858

[64] Wu Q, Wang X, Wan D, et al. Crosstalk of JNK1-STAT3 is critical for RAW264.7 cell survival. Cell Signal 2014;26:2951–60. 25269780 10.1016/j.cellsig.2014.09.013

[65] Cicenas J, Zalyte E, Rimkus A, et al. JNK, p38, ERK, and SGK1 Inhibitors in Cancer. Cancers 2017;10:1. 10.3390/cancers10010001 PMC5789351 29267206

[66] Montagut C, Settleman J. Targeting the RAF-MEK-ERK pathway in cancer therapy. Cancer Lett 2009;283:125–34. 19217204 10.1016/j.canlet.2009.01.022

[67] Meng L, Huang Z. In silico-in vitro discovery of untargeted kinase-inhibitor interactions from kinase-targeted therapies: a case study on the cancer MAPK signaling pathway. Comput Biol Chem 2018;75:196–204. 29803964 10.1016/j.compbiolchem.2018.05.012

[68] Bennett BL, Sasaki DT, Murray BW, et al. SP600125, an anthrapyrazolone inhibitor of Jun N-terminal kinase. Proc Natl Acad Sci U S A 2001;98:13681–6. 11717429 10.1073/pnas.251194298 PMC61101

[69] Zhang XJ, He C, Li P, et al. Ginsenoside Rg1, a potential JNK inhibitor, protects against ischemia/reperfusion-induced liver damage. J Funct Foods 2015;15:580–92.

[70] Grassi ES, Vezzoli V, Negri I, et al. SP600125 has a remarkable anti-cancer potential against undifferentiated thyroid cancer through selective action on ROCK and p53 pathways. Oncotarget 2015;6:36383–99. 26415230 10.18632/oncotarget.5799 PMC4742184

[71] Cerbone A, Toaldo C, Pizzimenti S, et al. AS601245, an anti-inflammatory JNK inhibitor, and clofibrate have a synergistic effect in inducing cell responses and in affecting the gene expression profile in CaCo-2 colon cancer cells. PPAR Res 2012;2012:1–16. 10.1155/2012/269751 PMC3349252 22619672

[72] Cerbone A, Toaldo C, Minelli R, et al. Rosiglitazone and AS601245 decrease cell adhesion and migration through modulation of specific gene expression in human colon cancer cells. PLoS One 2012;7:e40149. 22761953 10.1371/journal.pone.0040149 PMC3386191

[73] Ebelt ND, Kaoud TS, Edupuganti R, et al. A c-Jun N-terminal kinase inhibitor, JNK-IN-8, sensitizes triple negative breast cancer cells to lapatinib. Oncotarget 2017;8:104894–912. 29285221 10.18632/oncotarget.20581 PMC5739608

[74] Zhang T, Inesta-Vaquera F, Niepel M, et al. Discovery of potent and selective covalent inhibitors of JNK. Chem Biol 2012;19:140–54. 22284361 10.1016/j.chembiol.2011.11.010 PMC3270411

[75] Flemming A. Fungal infection: JNK inhibitors boost antifungal immunity. Nat Rev Immunol 2017;17:149. 28239167 10.1038/nri.2017.18

[76] Okada M, Kuramoto K, Takeda H, et al. The novel JNK inhibitor AS602801 inhibits cancer stem cells in vitro and in vivo. Oncotarget 2016;7:27021–27032. 27027242 10.18632/oncotarget.8395 PMC5053629

[77] Orci LA, Lacotte S, Oldani G, et al. The role of hepatic ischemia-reperfusion injury and liver parenchymal quality on cancer recurrence. Dig Dis Sci 2014;59:2058–2068. 24795038 10.1007/s10620-014-3182-7

[78] Jiang W, Wang Z, Jiang Y, et al. Ginsenoside Rg1 ameliorates motor function in an animal model of Parkinson’s Disease. Pharmacology 2015;96:25–31. 26065578 10.1159/000431100

[79] Prasad KD, Trinath J, Biswas A, et al. Alkyl chain substituted 1,9-pyrazoloanthrones exhibit prominent inhibitory effect on c-Jun N-terminal kinase (JNK). Org Biomol Chem 2014;12:4656–62. 24853961 10.1039/c4ob00548a

[80] Angell RM, Atkinson FL, Brown MJ, et al. N-(3-Cyano-4,5,6,7-tetrahydro-1-benzothien-2-yl)amides as potent, selective, inhibitors of JNK2 and JNK3. Bioorg Med Chem Lett 2007;17:1296–1301. 17194588 10.1016/j.bmcl.2006.12.003

[81] Jang WY, Lee JY, Lee ST, et al. Inhibition of JNK2 and JNK3 by JNK inhibitor IX induces prometaphase arrest-dependent apoptotic cell death in human Jurkat T cells. Biochem Biophys Res Commun 2014;452:845–851. 25218503 10.1016/j.bbrc.2014.09.015

[82] Liu J, Liu A. Role of JNK activation in apoptosis: a double-edged sword. Cell Res 2005;15:36–42. 15686625 10.1038/sj.cr.7290262

[83] Borsello T, Clarke PG, Hirt L, et al. A peptide inhibitor of c-Jun N-terminal kinase protects against excitotoxicity and cerebral ischemia. Nat Med 2003;9:1180–6. 12937412 10.1038/nm911

[84] Stebbins JL, De SK, Machleidt T, et al. Identification of a new JNK inhibitor targeting the JNK-JIP interaction site. Proc Natl Acad Sci U S A 2008;105:16809–13. 18922779 10.1073/pnas.0805677105 PMC2567907

[85] Asano Y, Kitamura S, Ohra T, et al. Discovery, synthesis and biological evaluation of isoquinolones as novel and highly selective JNK inhibitors (1). Bioorg Med Chem 2008;16:4715–32. 18313304 10.1016/j.bmc.2008.02.027

[86] Yao K, Cho YY, Bode AM, et al. A selective small-molecule inhibitor of c-Jun N-terminal kinase 1. FEBS Lett 2009;583:2208–12. 19527717 10.1016/j.febslet.2009.06.017

[87] Manning AM, Davis RJ. Targeting JNK for therapeutic benefit: from junk to gold? Nat Rev Drug Discov 2003; 2:554–65. 12815381 10.1038/nrd1132

[88] Neitz RJ, Konradi AW, Sham HL, et al. Highly selective c-Jun N-terminal kinase (JNK) 3 inhibitors with in vitro CNS-like pharmacokinetic properties II. Central core replacement. Bioorg Med Chem Lett 2011;21:3726–9. 21570836 10.1016/j.bmcl.2011.04.074

[89] Swahn B-M, Huerta F, Kallin E, et al. Design and synthesis of 6-anilinoindazoles as selective inhibitors of c-Jun N-terminal kinase-3. Bioorg Med Chem Lett 2005;15:5095–9. 16140012 10.1016/j.bmcl.2005.06.083

[90] Swahn BM, Xue Y, Arzel E, et al. Design and synthesis of 20-anilino-4,40-bipyridines as selective inhibitors of c-Jun N-terminal kinase-3. Bioorg Med Chem Lett 2006;16:1397–401. 16337120 10.1016/j.bmcl.2005.11.039

[91] Zhao Y, Spigolon G, Bonny C, et al. The JNK inhibitor D-JNKI-1 blocks apoptotic JNK signaling in brain mitochondria. Mol Cell Neurosci 2012;49:300–10. 22206897 10.1016/j.mcn.2011.12.005

[92] Zheng K, Park CM, Iqbal S, et al. Pyridopyrimidinone derivatives as potent and selective c-Jun N-terminal kinase (JNK) inhibitors. ACS Med Chem Lett 2015;6:413–8. 25893042 10.1021/ml500474d PMC4394340

[93] Kersting S, Behrendt V, Kersting J, et al. The impact of JNK inhibitor D-JNKI-1 in a murine model of chronic colitis induced by dextran sulfate sodium. J Inflamm Res 2013;6:71–81. 23667316 10.2147/JIR.S40092 PMC3650567

[94] Plantevin Krenitsky V, Nadolny L, Delgado M, et al. Discovery of CC-930, an orally active anti-fibrotic JNK inhibitor. Bioorg Med Chem Lett 2012; 22:1433–8. 22244937 10.1016/j.bmcl.2011.12.027

[95] Karin M, Gallagher E. From JNK to pay dirt: jun kinases, their biochemistry, physiology and clinical importance. IUBMB Life 2005;57:283–95. 16036612 10.1080/15216540500097111

[96] Gao YJ, Cheng JK, Zeng Q, et al. Selective inhibition of JNK with a peptide inhibitor attenuates pain hypersensitivity and tumor growth in a mouse skin cancer pain model. Exp Neurol 2009;219:146–55. 19445931 10.1016/j.expneurol.2009.05.006 PMC2728781

[97] Posthumadeboer J, van Egmond PW, Helder MN, et al. Targeting JNK-interacting-protein-1 (JIP1) sensitises osteosarcoma to doxorubicin. Oncotarget 2012;3:1169–81. 23045411 10.18632/oncotarget.600 PMC3717953

[98] Zhang H, Niu X, Qian Z, et al. The c-Jun N-terminal kinase inhibitor SP600125 inhibits human cytomegalovirus replication. J Med Virol 2015;87:2135–44. 26058558 10.1002/jmv.24286

[99] Beydoun T, Deloche C, Perino J, et al. Subconjunctival injection of XG-102, a JNK inhibitor peptide, in patients with intraocular inflammation: a safety and tolerability study. J Ocul Pharmacol Ther 2015;31:93–9. 25347151 10.1089/jop.2013.0247

[100] Desir S, O’Hare P, Vogel RI, et al. Chemotherapy-induced tunneling nanotubes mediate intercellular drug efflux in pancreatic cancer. Sci Rep 2018;8:9484. 29930346 10.1038/s41598-018-27649-x PMC6013499

[101] Gong L, Tan YC, Boice G, et al. Discovery of a novel series of 4-quinolone JNK inhibitors. Bioorg Med Chem Lett 2012;22:7381–7 23142618 10.1016/j.bmcl.2012.10.066

[102] He Y, Kamenecka TM, Shin Y, et al. Synthesis and SAR of novel quinazolines as potent and brain-penetrant c-jun N-terminal kinase (JNK) inhibitors. Bioorg Med Chem Lett 2011;21:1719–23. 21316221 10.1016/j.bmcl.2011.01.079 PMC3052630

[103] Ogura M, Uchida T, Terui Y, et al. Phase I study of OPB-51602, an oral inhibitor of signal transducer and activator of transcription 3, in patients with relapsed/refractory hematological malignancies. Cancer Sci 2015;106:896–901. 25912076 10.1111/cas.12683 PMC4520642

[104] Okusaka T, Ueno H, Ikeda M, et al. Phase 1 and pharmacological trial of OPB-31121, a signal transducer and activator of transcription-3 inhibitor, in patients with advanced hepatocellular carcinoma. Hepatol Res 2015;45:1283–91. 25676869 10.1111/hepr.12504

[105] Mahadevan D, Chiorean EG, Harris WB, et al. Phase I pharmacokinetic and pharmacodynamic study of the pan-PI3K/mTORC vascular targeted pro-drug SF1126 in patients with advanced solid tumours and B-cell malignancies. Eu J Cancer 2012;48:3319–27. 10.1016/j.ejca.2012.06.027 PMC3826796 22921184

[106] Makker V, Recio FO, Ma L, et al. A multicenter, single-arm, open-label, phase 2 study of apitolisib (GDC-0980) for the treatment of recurrent or persistent endometrial carcinoma (MAGGIE study). Cancer 2016;122:3519–28. 27603005 10.1002/cncr.30286 PMC5600677

[107] Brown JR. How I treat CLL patients with ibrutinib. Blood 2018;131:379–86. 29255067 10.1182/blood-2017-08-764712

